# Hydrogen sulfide improves vessel formation of the ischemic adductor muscle and wound healing in diabetic *db/db* mice

**DOI:** 10.22038/ijbms.2019.36551.8709

**Published:** 2019-10

**Authors:** Guo-Guang Wang, Wei Li

**Affiliations:** 1 Department of Pathophysiology, Wannan Medical College, Wuhu, China

**Keywords:** Adductor muscle Angiogenesis, db/db mice, Diabetes, Hydrogen sulfide, Ischemia

## Abstract

**Objective(s):**

It has been demonstrated that hydrogen sulfide plays a vital role in physiological and pathological processes such as regulating inflammation, oxidative stress, and vessel relaxation. The aim of the study was to explore the effect of hydrogen sulfide on angiogenesis in the ischemic adductor muscles of type 2 diabetic *db/db* mice and ischemic diabetic wound healing.

**Materials and Methods:**

The femoral arteries of diabetic *db/db* mice were isolated and ligated for preparation of ischemic hind limb model. Round incision was made on ischemic and non-ischemic limbs. The wounds were treated with sodium bisulfide (hydrogen sulfide donor). Real-time PCR and Western blotting were used to measure transcription of vascular endothelial growth factor (VEGF), epidermal growth factor (EGF), platelet derived growth factor (PDGF), hypoxia inducible factor-1α (HIF-1α) and endothelial nitric oxide synthase (eNOS) and protein expression of VEGF, VEGF receptor (VEGFR) and PDGF, PDGF receptor (PDGFR), respectively. Angiogenesis and morphological changes in adductor muscles were observed.

**Results:**

Hydrogen sulfide significantly increased transcription of VEGF, EGF, PDGF, HIF-1α, eNOS and protein expression of VEGF, PDGF, and phosphorylated VEGFR and PDGFR. Treatment with hydrogen sulfide significantly improved ischemic wound healing and formation of granulation tissue, and increased the number of small vessels in the ischemic adductor muscles.

**Conclusion:**

Our data suggested that hydrogen sulfide attenuated injury of ischemic adductor muscle, and promoted the ischemic diabetic wound healing via modulating angiogenesis in type 2 diabetic *db/db* mice.

## Introduction

Diabetes is increasingly becoming a worldwide problem affecting human health. Vascular diseases are the major complications of diabetes, which is the important factor of morbidity and mortality in patients with diabetes (1). Diabetic vascular lesion can implicate other vital organs such as the heart, kidney, and nerve (2-4). Diabetic foot ulcer (DFU) is one of the most fearing, and costly diabetic vascular complications due to its particular difficulty to heal, which is the first cause of nontraumatic lower extremity amputations (5). The pathogenesis of DFU is complex and still far from being fully understood, but diabetic vascular lesions are the dangerous factor for its development and nontraumatic amputation of lower limb in diabetic patients (6). Some studies have demonstrated that angiogenesis and vasculogenesis are beneficial to wound healing (7, 8). Vessel formation is a complex process, which relies on the interaction of various growth factors and cells (9). It is well-known that the expression of proangiogenic factors such vascular endothelial growth factor (VEGF) and platelet-derived growth factor (PDGF) plays a vital role in vessel formation, various experiments confirmed that VEGF and PDGF improve vessel formation, and diabetic wound healing (10, 11). Increasing results indicated that hyperglycemia reduces VEGF and PDGF expression in diabetic wound and impairs vessel formation, which delays diabetic wound healing (12, 13). Therefore, angiogenesis and vasculogenesis may be beneficial to the diabetic wound healing.

Hydrogen sulfide (H_2_S) was known as a toxic environmental pollutant with a terrible odor of rotten eggs (14), but it caught researchers’ interesting after endogenous hydrogen sulfide was discovered in brain in 1996 (15). Various studies showed that hydrogen sulfide plays a vital role in physiological and pathological processes such as regulating inflammation, oxidative stress, and vessel relaxation (16-18). As a result, hydrogen sulfide, along with nitric oxide (NO) and carbon monoxide (CO), is regarded as a gaseous signaling molecule. Recent reports suggested that hydrogen sulfide is also involved in pathogenesis of many diseases including Alzheimer disease, and several heart diseases (19-23). 

Hydrogen sulfide has been reported to improve neoangiogenesis via peroxisome proliferators-activated receptor (PPAR)-γ/VEGF axis (24). Our previous study suggested that hydrogen sulfide promotes diabetic wound healing via its anti-inflammation and anti-oxidative stress, and improves angiogenesis in wound tissues (25). Therefore, we hypothesized that hydrogen sulfide ameliorated angiogenesis in ischemic adductor via regulating VEGF and PDGF signaling and wound healing in type 2 diabetic *db/db* mice. 

## Materials and Methods

***Animals and experimental protocol***

All experimental procedure obeyed the Guide for the Care and Use of Laboratory Animals of the Chinese National Institutes of Health. Type 2 diabetic *db/db* mice were obtained from Changzhou Cavens Labobratory Animal CO LTD (Changzhou, China), and bred in standard facility with 22 ^°^C room temperature, and a 12-hour day/night alternate. Animals were assigned to four groups (8 mice per group): ischemia control (I-C), ischemia treatment (I-T), nonischemia control (N-C), and nonischemia treatment (N-T). All mice had access to food and water *ad libitum*.

***Preparation of animal model and treatment***

Mice were anesthetized via intraperitoneal injection with 10% chloral hydrate (300 mg/Kg). After shaved, left hind limb was made an incision for exposure of the femoral artery. The femoral artery was carefully separated from the femoral nerve and vein, and ligated for preparation of lower limb ischemia model in the mice from the I-C and the I-T, while not ligated in the N-C and the N-T mice. The I-T and the N-T mice were treated with sodium bisulfide (hydrogen sulfide donor) (0.1 ml, 30 mmol·l^-1^) by injection of quadriceps and gastrocnemius. A round incision (6 mm in diameter) was prepared on the dorsal skin of the left hind limb. The wounds from the I-T and the N-T mice were treated with 3% sodium bisulfide plaster. The wound closure was acquired with a digital camera.

***Determination of VEGF and PDGF***

Serum levels of VEGF and PDGF were measured with enzyme linked immunosorbent assay (ELISA) kits (Hefei Bomei Biotechnology CO, LTD, China) by colorimetric methods according to manufacturer’s protocol. The optical density (OD) values of each well were recorded, and the standard curve was plotted for determination of VEGF and PDGF contents.

***Morphological analyses***

At the end of experiment, the wound tissues and adductor muscles were collected, and fixed in 4% formalin. After embedded in paraffin, fixed samples were cut 5-μm-thickness sections. The sections were deparaffinized, and stained with hematoxylin-eosin (HE) for histological examinations. To measure granulation tissue thickness in the wounds, the sections were stained with Massone trichrome.

***Analyses of immunohistochemistry***

Deparaffinized 5-μm-thickness sections were treated with sodium citrate buffer for antigenic retrieval. To inhibit endogenous peroxidase, sections were incubated with 3% hydrogen peroxide in phosphate buffer solution for 15 min, and followed by treatment with phosphate buffer solution containing bovine serum albumin for blockage of nonspecific sites. Then sections were incubated with PDGF, CD34 primary antibody (Santa Cruz Biotechnology, USA) overnight at 4 ^°^C. After rinsed with phosphate buffer solution, the sections were treated with biotin-conjugated secondary antibody for 15 min. The sections were washed with phosphate buffer solution, and hatched in streptavidin-horseradish peroxidase. 3, 3’-diaminobenzidine (DAB) was used to detect antigens by visualization.

***Real-time polymerase chain reaction (RT-PCR)***

RT-PCR was used to assess mRNA expressions of VEGF, PDGF, HF-1α, FGF2, and eNOS. Total RNA of adductor muscles was extracted with Trizol Reagent (Invitrogen, USA). Genomic DNA contamination was removed from the RNA with Dnase. RNA concentration was determined via detecting the absorbance at 260 nm wavelength. RNA was utilized to synthetize cDNA by reverse transcriptase kit. PCR primers and probes are listed in Table 1. β-actin mRNA was used to measure the relative expressions of target genes. The relative levels of gene were expressed as 2^-ΔΔCT^.

***Western blotting***

Adductor muscles (50 mg) were collected and lysed in precooled lysis buffer (1% Triton-X 100, 50mM HEPES, 10mM Na_3_VO_4_, 100 mM NaF, 100 mM Na_4_P_2_O_7_, 10 μg/l leupeptin and aprotinin, 2mmol/l phenylmethanesulfonyl fluoride) on ice for 20 min, and subsequently centrifuged at 12,000 g for 15 min at 4 ^°^C. Amount of protein in supernatant was determined with a BCA kit (Bio-Rad). Proteins in the supernatant were electrophoretically separated by sodium dodecylsulphate polyacrylamide gel electrophoresis (SDS-PAGE), and then transferred to nitrocellulose membranes. The membranes were immersed in 5% nonfat milk containing primary antibodies VEGF, VEGFR, ERK, p-ERK, PDGF, PDGFR, Akt, p-Akt, and β-actin (1 : 500, Abcam, Cambridge, UK) overnight at 4 ^°^C, respectively. After rinsed with PBS, membranes were incubated with a peroxidase-conjugated secondary antibody (1:10000, Sigma, USA) for 2 hr. Target proteins were determined with visualization by DAB. β-actin as an internal control protein was used in the experiment.

**Figure 1:**
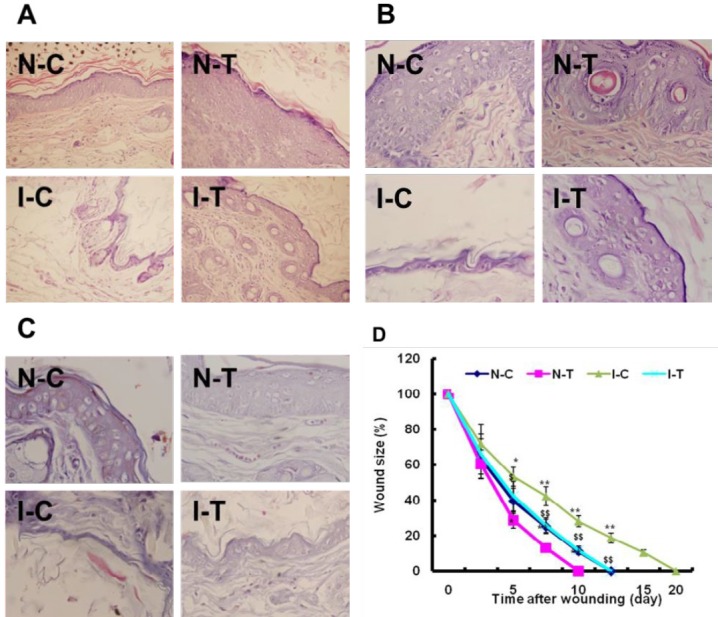
Effect of hydrogen sulfide on ischemic diabetic wound healing mice

(A, B) HE staining of wound tissues; (A) Magnification is 400×; (B) Magnification is 1000×; (C) Masson trichrome staining of wound tissues. Magnification is 400×; (D) Change of the wound. ^*^P-value<0.05, **P-value<0.01 compared with nonischemia control (N-C) group and ^$^*P-value*<0.05, ^$$^*P-value*<0.01 compared with ischemia control (I-C) group HE: Hematoxylin-eosin

**Figure 2:**
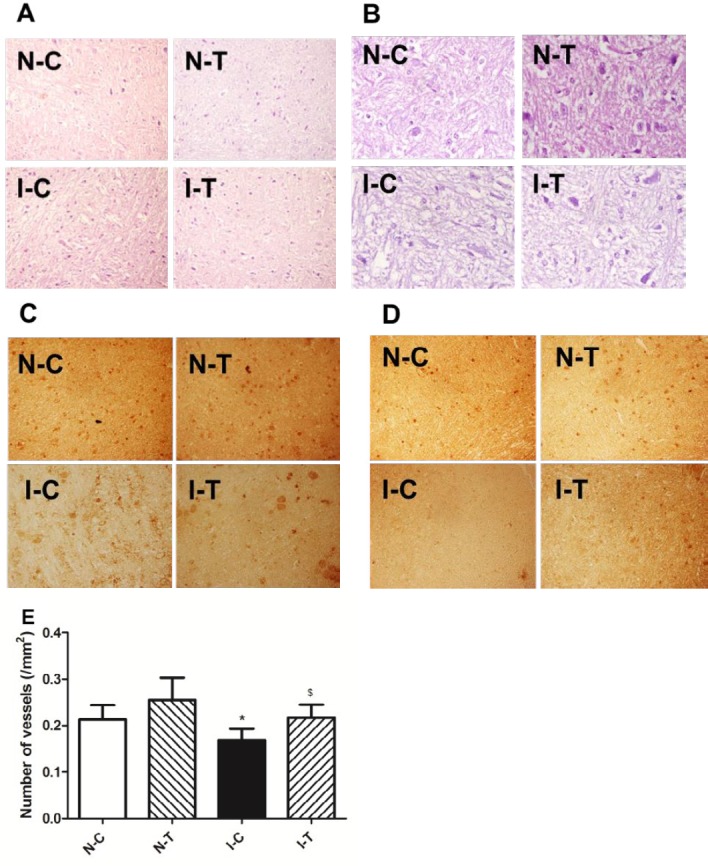
Histological analysis of ischemic adductor muscles

(A, B) HE staining of adductor muscles; (A) Magnification is 400×; (B) Magnification is 1000×; (C) Immunohistochemistry staining of CD34. Magnification is 400×; (D) Immunohistochemistry staining of PDGF. Magnification is 400×; (E) Quantification of vascular density in adductor muscles. ^*^P-value <0.05 compared with nonischemia control (N-C) group and ^$^*P-value* <0.05 compared with ischemia control (I-C) group

HE: Hematoxylin-eosin

**Figure 3:**
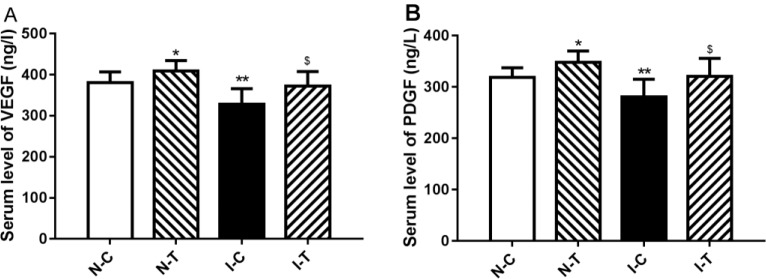
Serum levels of proangiogenic factors in ischemic diabetic and nonischemic mice

(A) VEGF concentration; (B) PDGF concentration. ^*^P-value <0.05, ^**^P-value <0.01 compared with nonischemia control (N-C) group and ^$^*P-value *<0.05, ^$$^*P-value* <0.01 compared with ischemia control (I-C) group

VEGF: Vascular endothelial growth factor; PDGF: Platelet derived growth factor

**Figure 4:**
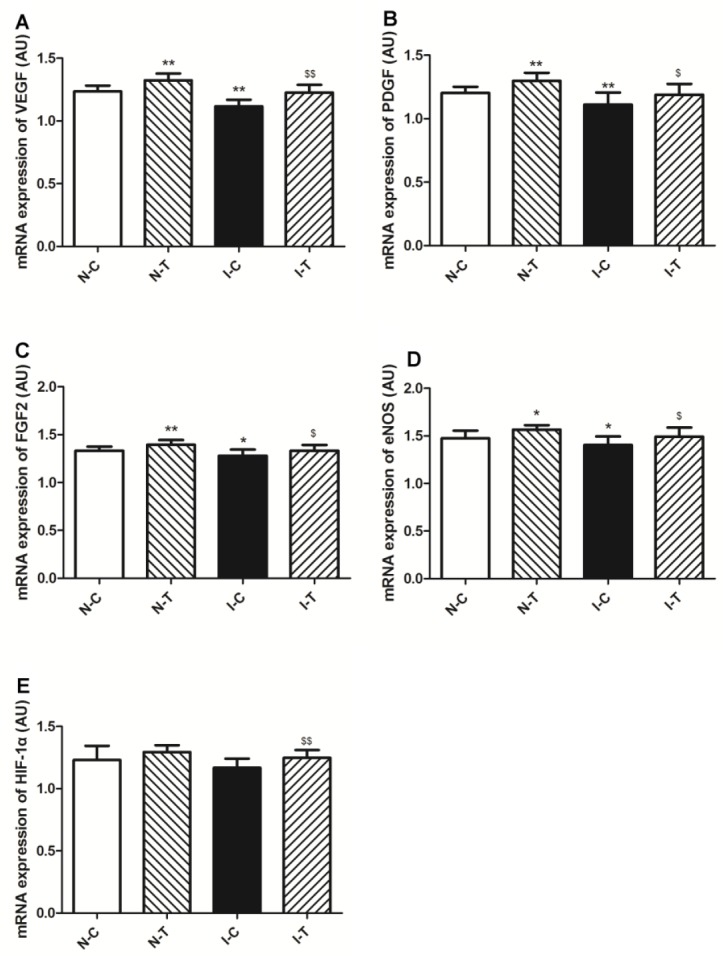
Gene expression of proangiogenic factors in ischemic diabetic and nonischemic adductor muscles

(A) VEGF Mrna level; (B) PDGF mRNA level; (C) eNOS mRNA level; (D) FGF2 mRNA level; (E) HIF-1α mRNA level. ^*^P-value <0.05, ^**^P-value <0.01 compared with nonischemia control (N-C) group and ^$^*P-value* <0.05, ^$$^*P-value* <0.01 compared with ischemia control (I-C) group

VEGF: Vascular endothelial growth factor; PDGF: Platelet derived growth factor

**Figure 5:**
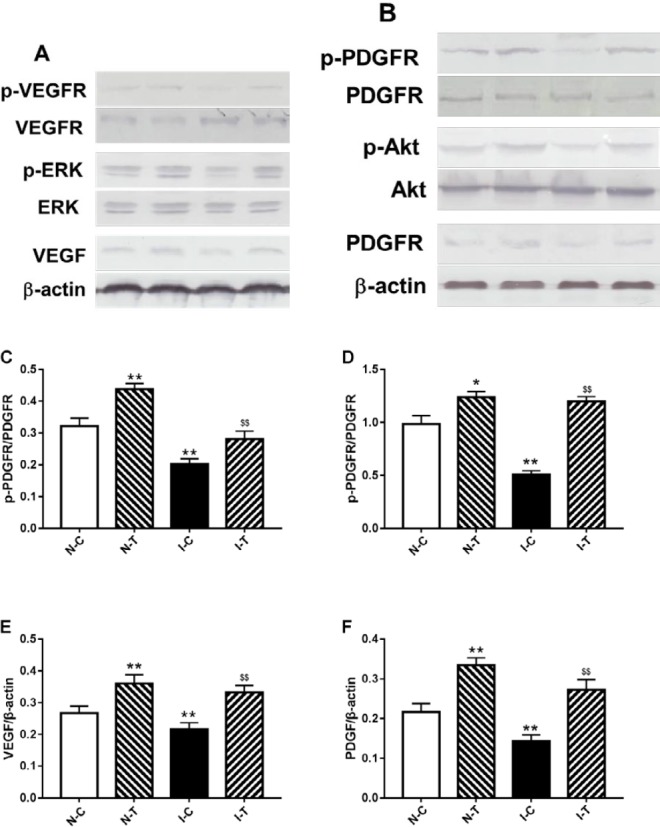
VEGF (A) and PDGF (B) signaling in ischemic diabetic and nonischemic adductor muscles. The relative density of protein expression levels of p-VEGFR (C), p-PDGFR (D), VEGF (E) and PDGF (F) in four studied groups analyzed by one-way ANOVA. Significant differences between groups are indicated by symbols (^**^P-value<0.01 compared with the N-C group and ^$$^*P-value*<0.01 compared with the I-C group)

VEGF: Vascular endothelial growth factor; PDGF: Platelet derived growth factor

**Table 1:** Sequence of primers

Gene	Forward	Reverse
VEGF	CACAGCAGATGTGAATGCAG	TTTACACGTCTGCGGATCTT
PDGF	CTCTTGGAGATAGACTCCGTAGG	ACTTCTCTTCCTGCGAATGG
HIF-1α	GGGTACAAGAAACCACCCAT	GAGGCTGTGTCGACTGAGAA
FGF-2	CAACCGGTACCTTGCTATGA	TCCGTGACCGGTAAGTATTG
PDGFR-β	ATCCGCTCCTTTGATGATCT	GAGCTTTCCAACTCGACTCC
eNOS	CCTTCCGCTACCAGCCAGA	CAGAGATCTTCACTGCATTGGCTA
ACTB	AGTGTGACGTTGACATCCGT	TGCTAGGAGCCAGAGCAGTA

***Statistical***

The data were presented as mean ± standard deviation (SD). Statistical differences between two groups were analyzed with one-way analysis of variance (*ANOVA*) followed by a *Tukey’s post hoc* analysis. GraphPad Prism (Version 7) was used for statistical analyses. A value of *P*<0.05 was considered to be statistically significant.

## Results

***Wound closure and histopathological observation***

The wounds were turgid and purulent, and there was no significant difference in the rate of the wound closure in the next two days. The rate of the wound closure was significantly decreased in the I-C mice compared with the N-C (*P*-value<0.05), and hydrogen sulfide caused a faster wound healing in the I-T than in the I-C mice (*P*-value<0.05) after 5 days (Figure 1).

Mature hair follicles and stratified epithelium were observed in wound sections stained from nonischemic mice, and epithelium was thicker, and more epidermis nipples and irregular collagen fiber were observed in wound sections from the untreated ischemic mice than in the treated ischemic. This suggested that hydrogen sulfide improved angiogenesis/neovascularization and the immigration of local fibroblasts, further accelerated ischemic wound healing (Figure 1). 

***Angiogenesis in adductor of ischemic hind limb***

Angiogenesis/vasculogenesis accelerates formation of granulation tissues by improvement of microcirculation and development of anti-infection in the wound. Therefore, capillary was observed in sections stained with H-E. The results showed that ischemia impaired angiogenesis, and the number of small vessels was reduced in ischemic adductors compared to nonischemic muscles. Treatment of hydrogen sulfide increased the number of capillaries in muscles of ischemic hind limbs. Further, IHC was used to observe expression of CD34 (a marker of neovascularization), expression of CD34 was reduced in ischemic adductors while hydrogen sulfide increased expression of CD34 in ischemic muscles (Figure 2).

***Changes of proangiogenic growth factors in adductor of ischemic hind limb***

Serum levels of PDGF and VEGF were decreased in the I-C mice compared to the N-C (*P*-value<0.01). Hydrogen sulfide treatment increased levels of PDGF and VEGF in the I-T mice compared with the I-C (*P*-value<0.05) in serum (Figure 3), and serum levels of PDGF and VEGF were significantly increased in the N-T mice compared with the N-C (*P*-value<0.05).

To further investigate the mechanism of hydrogen sulfide on angiogenesis, proangiogenic growth factor mRNA expression was determined in adductor of ischemic hind limb. The results of RT-PCR determination revealed that ischemia significantly decreased expressions of VEGF (*P*-value<0.01), FGF-2 (*P*-value<0.05), PDGF (*P*-value<0.01), eNOS (*P*-value<0.05) mRNA in adductors of ischemic limb compared with nonischemic adductors (Figure 4). Hydrogen sulfide treatment significantly increased expression of VEGF (*P*-value<0.01), FGF-2 (*P*-value<0.05), PDGF (*P*-value<0.05), eNOS (*P*-value<0.05) mRNA in ischemic muscles (Figure 4), and mRNA expression of these proangiogenic growth factors was significantly increased in the N-T mice compared with the N-C (Figure 4). HIF-1α mRNA expression was not significantly changed in the I-C mice compared with the N-C (*P*-value>0.05), but hydrogen sulfide significantly increased expression of HIF-1α mRNA in ischemic adductor (*P*-value<0.05) rather than nonischemic adductor (*P*-value>0.05) (Figure 4).

Analysis of immunohistochemistry showed that expressions of PDGF and VEGF protein were decreased in ischemic muscles compared to the nonischemic, and hydrogen sulfide improved expressions of PDGF and VEGF protein (Figure 5).

***Activation of angiogenesis signaling pathway in adductor of ischemic hind limb***

To further observe the mechanisms of hydrogen sulfide promoting angiogenesis, VEGF and PDGF protein expression and their respective signaling pathway were detected. Expression of VEGF and PDGF protein expression was decreased in ischemic versus nonischemic adductor muscles, and phosphorylation of their receptors (VEGFR and PDGFR), along with ERK and Akt phosphorylation was reduced in the ischemic muscles. Hydrogen sulfide increased phosphorylation of VEGFR, PDGF, ERK and Akt in the I-C mice compared with the I-T mice (Figure 5).

## Discussion

Sustained hyperglycemia resulting in diabetes is implicated in progression of vascular diseases. Vascular complication is a vital risk factor and the primary cause for nontraumatic lower extremity amputations. In present study, our results showed that ischemia delays cutaneous wound healing in Type 2 diabetic *db/db* mice, abates the expression of vital proangiogenic factors such as VEGF and PDGF, decreases phosphorylation of their receptors, and impairs angiogenesis/vasculogenesis in ischemic muscles. Hydrogen sulfide restrains the decline of VEGF and PDGF expression, restores phosphorylation of PDGFR and VEGFR, and improves capillary formation and ischemic wound healing.

Wound healing is a complex and elaborate pathophysiological process, which implicates a cooperative and accurate interaction of various growth factors and cells. Loss of blood supply impaired wound repair and rehabilitation. Therefore, neovascularization favors formation of granulation tissues which are essential to wound healing, and provides various growth factors and nutriment for tissue repair (26-28). Hydrogen sulfide, a gas signaling molecule, was found to play an important role in various physiological events such as regulating vasorelaxation and hemodynamics (29, 30). Further, hydrogen sulfide is implicated in mediating proliferation and migration of endothelial cells, which accelerates vessel formation (31, 32). In present study, our results showed that ischemia delayed diabetic wound healing, and impaired vessel formation in ischemic adductor muscles. Hydrogen sulfide promoted ischemic wound healing, and epithelium was thicker in the wound while more capillaries were observed in ischemic adductor muscles. Collagen fiber was more regular in the wound treated with hydrogen sulfide. Therefore, hydrogen sulfide may increase new vessel formation and migration and proliferation, which synergistically accelerate diabetic ischemic wound healing.

Proangiogenic factors including VEGF and PDGF contribute to angiogenesis. VEGF has been reported to improve angiogenesis via regulating migration and proliferation of endothelial cells (33-35). Animal experiments showed that PDGF increases collateral vessel formation (36). It is well-known that expression of VEGF and PDGF is decreased in ischemia, hypoxia and diabetes (9, 12, 13). Several studies in animals indicated that increased expression of VEGF and PDGF improves angiogenesis, diabetic wound healing (11, 37-39). HIF-1α is a vital regulator of proangiogenic factors in hypoxic tissues. Increased expression of HIF-1α enhances eNOS activation, and promotes angiogenesis (40). Our study showed that expression of proangiogenic factor genes such as VEGF, PDGF, eNOS and EGF was decreased in ischemic adductor muscles. Hydrogen sulfide increased expression of these genes. Interestingly, expression of HIF-1α gene was decreased but not statistical significance in the ischemic adductor muscles compared with the nonischemic adductor, but hydrogen sulfide treatment significantly increased expression of HIF-1α mRNA in ischemic adductor muscles.

Proangiogenic factors play a vital role in angiogenesis, but administration of VEGF via gene therapy has not presented the long-term benefits (41). Therefore, clinical effects of these growth factors were limited (42). Further studies documented that impairment of these factor signaling, rather than their expression, disrupts angiogenesis and diabetic wound repair (43, 44). Our data showed that phosphorylation of VEGFR and PDGFR, along with Akt and ERK was reduced in ischemic adductor muscles compared with the nonischemic, and hydrogen sulfide increased phosphorylation of these proteins. These suggested that hydrogen sulfide promotes angiogenesis and diabetic wound healing via improving VEGFR and PDGFR signaling.

## Conclusion

Our data revealed that hydrogen sulfide promotes wound healing in ischemic diabetic lower limb, increases expression of proangiogenesis factors and vessel formation in ischemic adductor muscles. Our results suggested that hydrogen sulfide may provide a novel potential strategy toward developing it a valid therapeutic choice for chronic diseases associated with failure of angiogenesis.
